# L-shaped association between serum chloride levels with 90-day and 365-day all-cause mortality in critically ill patients with COPD: a retrospective cohort study

**DOI:** 10.1038/s41598-024-67008-7

**Published:** 2024-07-10

**Authors:** Shidong Wang, Dai Li, Yaokun Wang, Linmin Lu, Xiaoyan Hu, Weibiao Wang

**Affiliations:** Department of Respiratory Medicine, Shaoxing Second Hospital, Zhejiang, China

**Keywords:** Serum chloride, COPD, All-cause mortality, Respiratory tract diseases, Chronic obstructive pulmonary disease, Predictive markers, Prognostic markers

## Abstract

This study aimed to investigate the association between serum chloride levels and all-cause mortality in critically ill patients with chronic obstructive pulmonary disease (COPD). Data from the Medical Information Mart for Intensive Care IV (MIMIC-IV) database were extracted for analysis. Demographic information, laboratory results, medical histories, vital signs, and prognosis-related data were collected. Cox proportional hazard models were used to assess the relationship between serum chloride levels and 90-day and 365-day mortality. Subgroup analyses were conducted to explore potential interactions between serum chloride levels and various factors. The study included patients with a median age of 72.00 years, of whom 52.39% were male. Higher quartiles of serum chloride levels were associated with significantly lower levels of weight, RBC, platelet, hemoglobin, and other variables (*P* < 0.05), accompanied by lower 90-day and 365-day mortality (*P* < 0.05). Cox proportional hazard model indicated that the risk of death was significantly lower in the fourth quartile of serum chloride levels compared with the first quartile after adjusting for confounders (90-day HR = 0.54, 365-day HR = 0.52, both *P* < 0.05). An L-shape relationship was observed, with risks of death decreasing as serum chloride levels increased, although the magnitude decreased when levels reached 102 mmol/L. This study demonstrated an independent L-shaped association between serum chloride levels and all-cause mortality in critically ill patients with COPD. This finding helps us to understand the prognostic value of serum chloride levels in critically ill patients with COPD.

## Introduction

Chronic obstructive pulmonary disease (COPD) is a prevalent lung disease characterized by airway obstruction, inflammation, and alveolar destruction. This leads to symptoms such as dyspnea, cough, and sputum production. The high prevalence of COPD is a significant public health concern, with epidemiological surveys in the United States indicating an approximate 6.0% prevalence among adults over the past decade^1^. Importantly, COPD exhibits notable age-related disparities in both incidence and mortality, with higher rates observed among the elderly population compared to the young population. Notably, COPD ranks as the third leading cause of global incidence and the sixth in the United States, making it a common disease encountered in intensive care units (ICU)^2^. According to a retrospective cohort study, critically ill patients with COPD face a significantly elevated risk of mortality compared to individuals without COPD^3^. This finding underscores the gravity of COPD as a risk factor for mortality in the critically ill population. Additionally, a study of patients with ventilator-associated pneumonia in the ICU showed increased mortality if they had COPD, further emphasizing the importance of COPD as a risk factor^4^. Nonetheless, limited research has specifically explored the risk of ICU mortality in COPD patients.

Serum chloride, as the primary extracellular ion in the human body, is a frequently measured biochemical parameter in clinical practice. In recent years, an increasing number of studies has explored the association between serum chloride levels and the incidence and mortality of various diseases. Particularly in the field of cardiovascular diseases, including congestive heart failure, hypertension, vascular inflammation, and myocardial disease, research has demonstrated a close relationship between serum chloride and these conditions^5–7^. For instance, a cohort study investigating late-stage decompensated heart failure patients revealed that serum chloride levels had a higher predictive value for mortality compared to serum sodium levels^5^. Similarly, in respiratory conditions, such as amyotrophic lateral sclerosis patients with respiratory failure, serum chloride levels have been observed to be associated with disease severity^8^. However, the specific relationship between serum chloride levels and mortality in patients with COPD, the most prevalent respiratory disease, has not been well-established. To address this knowledge gap, further investigation is warranted.

In this study, we aimed to investigate the association between serum chloride levels and mortality in critically ill patients with COPD. To achieve this objective, we used data from the Medical Information Mart for Intensive Care IV (MIMIC-IV) database. By utilizing this extensive database, we sought to shed light on the relationship between serum chloride levels and mortality in this specific population.

## Methods

### Data sources and population

This study employed a retrospective cohort design, utilizing the data extracted from the comprehensive MIMIC-IV database. This database comprises information collected from adult patients admitted to the intensive care units of the Beth Israel Deaconess Medical Center (BIDMC) in Boston, Massachusetts, from 2008 to October 2019^9^. Ethical considerations regarding patient privacy and confidentiality were addressed through the process of de-identification. The access to the database and subsequent data extraction was conducted by one of the authors, Shidong Wang, who has successfully completed a collaborative institution training program examination (certification number: 12114387). As this is a publicly available database, researchers who obtain the corresponding credentials are authorized to use it. At the same time, the ethics committee (The Institutional Review Board of the Massachusetts Institute of Technology and Beth Israel Deaconess Medical Center) had approved the database when it was established, so there was no need to obtain informed consent again for this study. This study followed and complied with the guidelines for cross-sectional studies established by Strengthening the Reporting of Observational Research in Epidemiology (STROBE).

**Figure 1:**
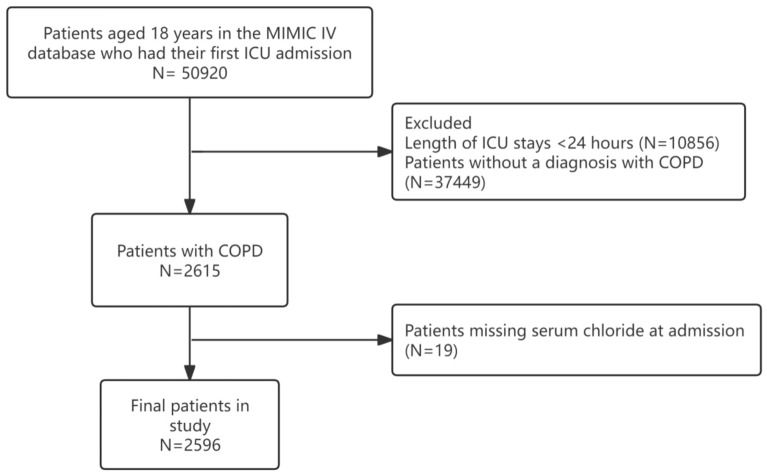
Flowchart of our study. COPD: chronic obstructive pulmonary disease.

A total of 50,920 participants were included in the study. The inclusion criteria for this study were: (1) patients who were admitted to the ICU for the first time and were over 18 years old, (2) patients who stayed in the ICU for at least 24 h, (3) patients diagnosed with COPD based on ICD-9 or ICD-10, and (4) patients without missing serum chloride indicators (Fig. 1).

### Data extraction

Data extraction for this study was conducted using pgAdmin 4 (version 7.8) and the SQL code for extracting variable information from the MIMIC database was taken from the official repository (https://github.com/MIT-LCP/mimic-code). The extracted data encompassed various aspects, including demographic information, laboratory results, medical history, vital signs, and prognosis-related data. The demographic information comprised the age, gender, and weight of the patients. Laboratory information encompassed white blood cells (WBC), red blood cells (RBC), platelets, hemoglobin, red cell distribution width (RDW), sodium, phosphate, calcium, glucose, prothrombin time (PT), partial thromboplastin time (PTT), blood urea nitrogen (BUN), serum creatinine (sCR), and chloride levels. The medical history section incorporated conditions such as hypertension, diabetes, heart failure, myocardial infarction, chronic kidney disease (CKD), stroke, and hyperlipidemia. Vital signs captured the systolic and diastolic blood pressure (SBP, DBP) readings. Additionally, data pertaining to disease scoring, such as sequential organ failure assessment (SOFA), acute physiology score III (APS III), Systemic Inflammatory Response Syndrome (SIRS), and Simplified Acute Physiology Score II (SAPS II), were also extracted. Serum chloride was stratified into quartiles, specifically Q1 (< 98.4 mmol/L), Q2 (98.4–102.0 mmol/L), Q3(102.0–106.0 mmol/L), and Q4 (> 106.0 mmol/L). The primary outcome measures for this study were the 90-day mortality and the 365-day mortality.

In Supplementary Table 1 the missing variables of this study are presented. For variables with missing data less than 20%, the random forest algorithm was used for imputation^10^. For variables with missing data greater than 20% but less than 40%, the variable was converted into a dummy variable. Variables with missing data exceeding 40% were not included in this study.

### Statistical analysis

Continuous variables that followed a normal distribution were represented using mean (standard deviation), while those with a skewed distribution were described using median and interquartile range. T-tests and ANOVA were employed for statistical analysis of normally distributed continuous variables, while the Kruskal–Wallis test was used for skewed continuous variables. Categorical variables were presented as numbers (percentages), and the chi-square test was used for analysis. Kaplan–Meier curves were utilized to depict the relationship between serum chloride levels and overall mortality at 90 and 365 days in critically ill COPD patients. To assess multicollinearity, the variance inflation factor (VIF) is computed. Variables that were clinically significant or had a *P*-value < 0.05 in univariate Cox regression with a VIF less than 5 were included in the multivariable Cox proportional hazards model. Four models, namely Model 1 (no adjusted), Model 2 (adjusted for age, gender, and weight), Model 3 (adjusted for age, gender, weight, WBC, RBC, platelet, hemoglobin, RDW, hypertension, diabetes, and heart failure), and Model 4 (adjusted for age, gender, weight, WBC, RBC, platelet, hemoglobin, RDW, hypertension, diabetes, and heart failure, myocardial infarction, CKD, stroke, hyperlipidemia, sodium, potassium, calcium, glucose, PT, PTT, BUN, sCR, SOFA, APS III, SIRS, SAPS II, SBP, and DBP), were established. Considering the potential for a non-linear relationship between serum chloride and overall mortality, restricted cubic splines (RCS) were employed to characterize how all-cause mortality changes with the variation of serum chloride levels. Subgroup analysis was conducted to compare the association between serum chloride and overall mortality at 90 and 365 days in critically ill COPD patients with different characteristics. Finally, we performed a sensitivity analysis to verify the robustness of the results.

All statistical analyses are performed using R software (version 4.1.3), with a *P*-value < 0.05 considered statistically significant.

### Ethical approval

The establishment of this database and this study was approved by the Massachusetts Institute of Technology (Cambridge, MA) and Beth Israel Deaconess Medical Center (Boston, MA), and consent was obtained for the original data collection.

## Results

### Baseline characteristics

Table 1 presents the baseline characteristics of the study subjects. The median age of critically ill COPD patients included in the study was 72.00 years, with 52.39% being male. Comparatively, patients in the highest quartile of serum chloride levels exhibited significantly lower levels of weight (*P* = 0.001), RBC (*P* < 0.001), platelet (*P* < 0.001), hemoglobin (*P* < 0.001), RDW (*P* < 0.001), potassium (*P* = 0.011), calcium (*P* < 0.001), glucose (*P* < 0.001), PT (*P* = 0.016), PTT (*P* < 0.001), BUN (*P* < 0.001), sCR (*P* < 0.001), APS III (*P* < 0.001), SBP (*P* = 0.004), and DBP (*P* < 0.001), when compared to those in the lowest quartile. Furthermore, patients in the higher quartile demonstrated lower 90-day and 365-day mortality, as well as reduced rates of heart failure and diabetes (both *P* < 0.001). Table 2 presents the characteristics of patients with different outcomes at 365 days. Factors such as age, weight (*P* < 0.001), WBC (*P* = 0.021), RBC (*P* < 0.001), hemoglobin (*P* < 0.001), BUN (*P* < 0.001), and SOFA (*P* < 0.001) were identified to be closely associated with the participants' outcomes.

**Table 1:** Baseline characteristics of participants grouped according to different serum chloride.

	Serum chloride					P
	Total (n = 2596)	Q1 (n = 639)	Q2 (n = 625)	Q3 (n = 673)	Q4 (n = 659)	
Age, years	72.00 (64.00–80.00)	72.00 (63.00–80.00)	71.00 (63.00–80.00)	72.00 (64.00–79.00)	73.00 (66.00–81.50)	0.013
Gender, n (%)						0.189
Male	1360 (52.39)	313 (48.98)	343 (54.88)	359 (53.34)	345 (52.35)	
Female	1236 (47.61)	326 (51.02)	282 (45.12)	314 (46.66)	314 (47.65)	
Weight, kg	78.38 (65.00–95.11)	80.00 (64.00–100.00)	80.00 (65.00–96.90)	78.60 (65.60–94.50)	75.80 (63.40–89.80)	0.001
WBC, K/µL	11.50 (8.40–15.28)	10.80 (7.97–14.48)	11.40 (8.10–15.23)	11.51 (8.47–15.60)	12.08 (9.25–15.77)	< 0.001
RBC, K/µL	3.49 (3.04–4.01)	3.62 (3.06–4.11)	3.57 (3.07–4.09)	3.52 (3.10–4.01)	3.35 (2.97–3.74)	< 0.001
Platelet, K/µL	189.50 (140.46–253.00)	203.50 (148.12–282.00)	198.50 (148.00–261.67)	188.50 (143.75–240.00)	167.67 (129.79–224.17)	< 0.001
Hemoglobin, g/dL	10.38 (8.90–11.80)	10.40 (8.91–12.09)	10.60 (9.00–12.10)	10.55 (9.13–11.90)	10.00 (8.80–11.27)	< 0.001
RDW, %	14.75 (13.70–16.30)	15.00 (13.91–16.90)	14.80 (13.77–16.35)	14.48 (13.50–16.05)	14.75 (13.60–16.02)	< 0.001
Sodium, mmol/L	139.00 (136.00–141.33)	135.00 (131.67–138.33)	138.00 (135.67–140.00)	139.33 (137.33–141.50)	141.00 (139.00–143.50)	< 0.001
Potassium, mmol/L	4.28 (3.95–4.68)	4.30 (3.99–4.80)	4.30 (3.97–4.68)	4.27 (3.92–4.61)	4.23 (3.93–4.60)	0.011
Calcium, mmol/L	8.40 (8.00–8.80)	8.65 (8.19–9.02)	8.47 (8.00–8.80)	8.40 (8.00–8.80)	8.14 (7.80–8.50)	< 0.001
Glucose, mmol/L	130.00 (110.00–163.04)	140.00 (113.00–178.75)	134.00 (111.25–168.50)	127.50 (109.00–156.00)	125.00 (107.00–151.00)	< 0.001
PT, sec	13.90 (12.40–16.40)	14.00 (12.40–17.66)	13.88 (12.30–16.15)	13.75 (12.27–15.95)	13.98 (12.66–16.14)	0.016
PTT, sec	32.60 (28.10–43.16)	34.07 (28.48–47.81)	33.60 (28.45–43.83)	32.00 (27.80–42.05)	31.13 (27.98–38.93)	< 0.001
BUN, mg/dL	21.00 (14.73–34.00)	24.50 (16.41–42.05)	20.50 (14.00–31.00)	19.50 (14.00–30.33)	21.00 (14.12–35.67)	< 0.001
sCR, mg/dL	1.00 (0.70–1.47)	1.05 (0.70–1.86)	1.00 (0.70–1.40)	0.93 (0.70–1.32)	1.00 (0.77–1.50)	< 0.001
SOFA	4.00 (2.00–7.00)	4.00 (2.00–7.00)	4.00 (2.00–7.00)	4.00 (2.00–7.00)	5.00 (3.00–8.00)	< 0.001
APS III	42.00 (32.00–55.00)	46.00 (34.00–58.00)	42.00 (33.00–53.00)	40.00 (31.00–51.00)	42.00 (32.00–56.00)	< 0.001
SIRS	3.00 (2.00–3.00)	3.00 (2.00–3.00)	3.00 (2.00–3.00)	3.00 (2.00–3.00)	3.00 (2.00–3.00)	0.061
SAPS II	37.00 (30.00–45.00)	37.00 (29.00–45.00)	36.00 (29.00–44.00)	36.00 (29.00–44.00)	39.00 (32.00–47.50)	< 0.001
SBP, mmHg	114.55 (106.14–126.13)	116.57 (106.19–127.94)	115.56 (106.50–127.23)	114.86 (106.48–125.99)	112.04 (104.95–123.31)	0.004
DBP, mmHg	63.28 (57.32–70.00)	63.83 (57.56–70.61)	65.00 (59.27–71.79)	63.08 (57.75–70.08)	61.30 (55.55–68.00)	< 0.001
Hypertension, n (%)	1042 (40.14)	230 (35.99)	261 (41.76)	292 (43.39)	259 (39.30)	0.038
Diabetes, n (%)	860 (33.13)	230 (35.99)	215 (34.40)	224 (33.28)	191 (28.98)	0.047
Heart failure, n (%)	1134 (43.68)	356 (55.71)	268 (42.88)	271 (40.27)	239 (36.27)	< 0.001
Myocardial infarction, n (%)	334 (12.87)	87 (13.62)	74 (11.84)	90 (13.37)	83 (12.59)	0.776
CKD, n (%)	617 (23.77)	165 (25.82)	133 (21.28)	138 (20.51)	181 (27.47)	0.006
Stroke, n (%)	233 (8.98)	57 (8.92)	54 (8.64)	61 (9.06)	61 (9.26)	0.984
Hyperlipidemia, n (%)	1192 (45.92)	266 (41.63)	266 (42.56)	333 (49.48)	327 (49.62)	0.002
90-day mortality, n (%)	657 (25.31)	211 (33.02)	142 (22.72)	142 (21.10)	162 (24.58)	< 0.001
365-day mortality, n (%)	941 (36.25)	306 (47.89)	202 (32.32)	214 (31.80)	219 (33.23)	< 0.001

Continuous variables are expressed using medians (quartiles) and categorical variables are expressed using numbers (percentages).

WBC, white blood cell; RBC, red blood cell; RDW, red blood cell distribution; PT, prothrombin time; PTT, partial thromboplastin time; BUN, blood urea nitrogen; sCR, serum creatinine; SOFA, sequential organ failure assessment; APS III, acute physiology score III; SIRS, systemic inflammatory response syndrome; SAPS II, simplified acute physiology score II; SBP, systolic blood pressure; DBP, diastolic blood pressure; CKD, chronic kidney disease.

**Table 2:** Baseline characteristics of the Survivors and Non-survivors groups.

	Total (n = 2615)	Survivors (n = 1664)	Non-survivors (n = 951)	*P*
Age, years	72.00 (64.00–80.00)	70.00 (63.00–78.00)	76.00 (68.00–83.00)	< 0.001
Gender, n (%)				0.742
Male	1360 (52.39)	863 (52.15)	497 (52.82)	
Female	1236 (47.61)	792 (47.85)	444 (47.18)	
Weight, kg	78.38 (65.00–95.11)	80.90 (67.00–96.85)	74.80 (61.15–91.00)	< 0.001
WBC, K/µL	11.50 (8.40–15.28)	11.30 (8.30–15.00)	12.03 (8.50–15.90)	0.021
RBC, K/µL	3.49 (3.04–4.01)	3.59 (3.11–4.07)	3.34 (2.91–3.87)	< 0.001
Platelet, K/µL	189.50 (140.46–253.00)	189.00 (144.29–248.00)	191.50 (131.67–260.00)	0.661
Hemoglobin, g/dL	10.38 (8.90–11.80)	10.65 (9.25–12.07)	9.87 (8.58–11.40)	< 0.001
RDW, %	14.75 (13.70–16.30)	14.45 (13.47–15.70)	15.44 (14.25–17.15)	< 0.001
Sodium, mmol/L	139.00 (136.00–141.33)	139.00 (136.00–141.00)	139.00 (135.40–142.00)	0.476
Potassium, mmol/L	4.28 (3.95–4.68)	4.27 (3.95–4.63)	4.30 (3.95–4.75)	0.034
Calcium, mmol/L	8.40 (8.00–8.80)	8.40 (8.00–8.80)	8.40 (7.94–8.83)	0.708
Glucose, mmol/L	130.00 (110.00–163.04)	128.67 (109.00–159.00)	135.00 (111.33–171.67)	< 0.001
PT, sec	13.90 (12.40–16.40)	13.65 (12.30–15.60)	14.56 (12.65–17.90)	< 0.001
PTT	32.60 (28.10–43.16)	31.68 (27.90–40.38)	34.75 (28.80–47.55)	< 0.001
BUN, mg/dL	21.00 (14.73–34.00)	18.50 (13.00–27.67)	28.00 (18.00–45.00)	< 0.001
sCR, mg/dL	1.00 (0.70–1.47)	0.90 (0.70–1.30)	1.18 (0.80–1.93)	< 0.001
SOFA	4.00 (2.00–7.00)	4.00 (2.00–6.00)	6.00 (3.00–9.00)	< 0.001
APS III	42.00 (32.00–55.00)	39.00 (30.00–49.00)	50.00 (38.00–63.00)	< 0.001
SIRS	3.00 (2.00–3.00)	3.00 (2.00–3.00)	3.00 (2.00–3.00)	< 0.001
SAPS II	37.00 (30.00–45.00)	35.00 (28.00–42.00)	42.00 (34.00–51.00)	< 0.001
SBP, mmHg	114.55 (106.14–126.13)	115.10 (107.35–126.79)	113.50 (104.58–125.14)	< 0.001
DBP, mmHg	63.28 (57.32–70.00)	63.95 (58.05–70.74)	62.08 (55.83–68.84)	< 0.001
Hypertension, n (%)	1042 (40.14)	734 (44.35)	308 (32.73)	< 0.001
Diabetes, n (%)	860 (33.13)	546 (32.99)	314 (33.37)	0.844
Heart failure, n (%)	1134 (43.68)	636 (38.43)	498 (52.92)	< 0.001
Myocardial infarction, n (%)	334 (12.87)	187 (11.30)	147 (15.62)	0.002
CKD, n (%)	617 (23.77)	318 (19.21)	299 (31.77)	< 0.001
Stroke, n (%)	233 (8.98)	139 (8.40)	94 (9.99)	0.173
Hyperlipidemia, n (%)	1192 (45.92)	800 (48.34)	392 (41.66)	0.001

Continuous variables are expressed using medians (quartiles) and categorical variables are expressed using numbers (percentages).

WBC, white blood cell; RBC, red blood cell; RDW, red blood cell distribution; PT, prothrombin time; PTT, partial thromboplastin time; BUN, blood urea nitrogen; sCR, serum creatinine; SOFA, sequential organ failure assessment; APS III, acute physiology score III; SIRS, systemic inflammatory response syndrome; SAPS II, simplified acute physiology score II; SBP, systolic blood pressure; DBP, diastolic blood pressure; CKD, chronic kidney disease.

### Primary outcomes

**Figure 2:**
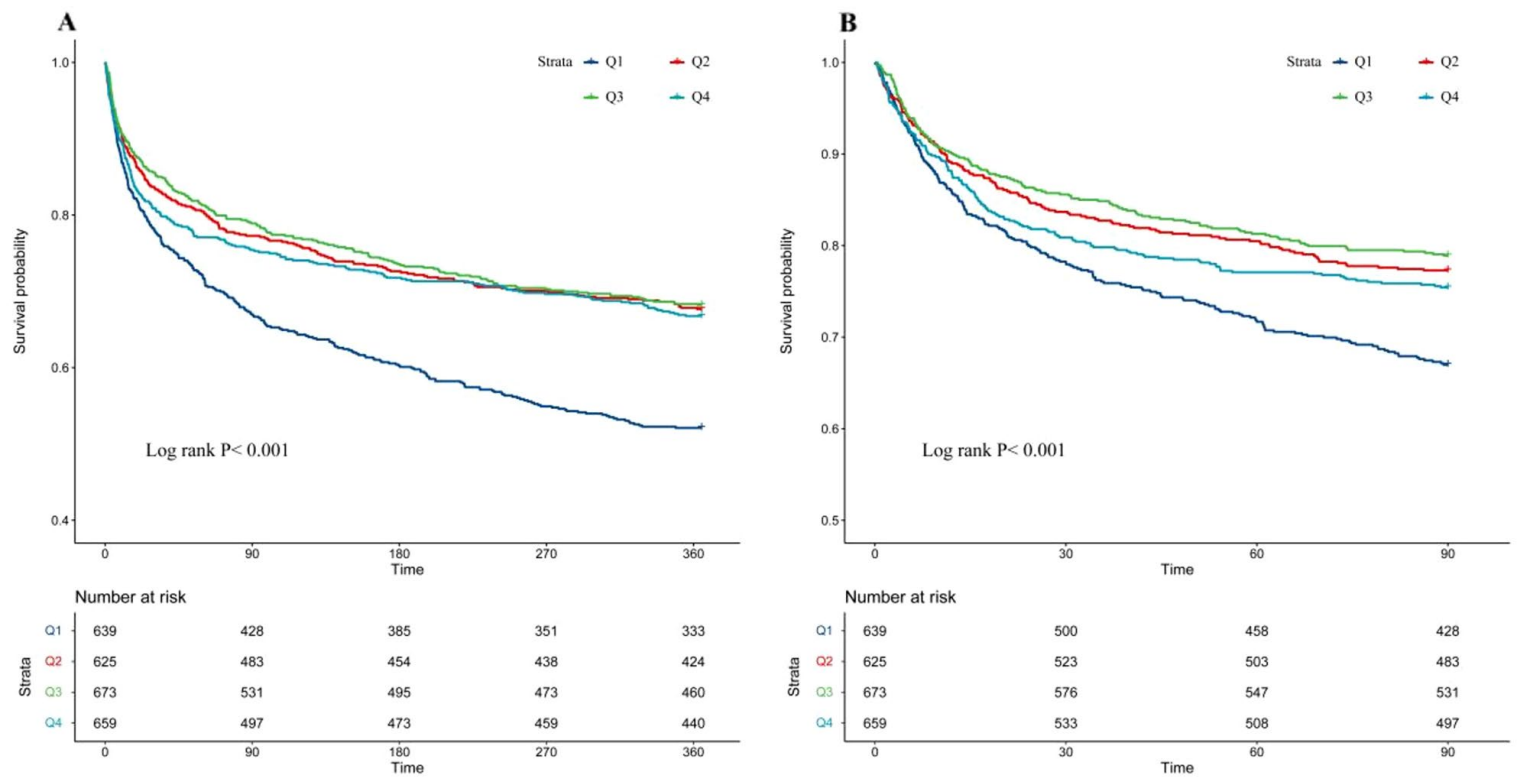
Kaplan–Meier survival analysis curve for all-cause mortality. ( A ) 365-day mortality, ( B ) 90-day mortality.

The Kaplan–Meier survival analysis curves displaying the main outcome rates among different groups based on quartiles of serum chloride levels are depicted in (Fig. 2). For the 90-day follow-up period, we observed a significant difference in survival probability between the four groups (log-rank *P* = 0.001). Specifically, patients in the lowest quartile (Q1) had a lower probability of survival, compared to patients in the highest quartile (Q4) who showed the highest probability of survival. Further, a similar pattern was found over the 365-day follow-up period (log-rank *P* < 0.001).

To further investigate the association between serum chloride levels and mortality in critically ill COPD patients, the Cox proportional hazards model was employed while adjusting for potential confounding factors (Table 3). The analysis demonstrated that after accounting for these factors, serum chloride levels remained significantly and independently associated with increased 90-day (HR = 0.96, 95%CI 0.94–0.98) and 365-day (HR = 0.96, 95%CI 0.94–0.97) mortality. Even when serum chloride was categorized into quartiles, the association between serum chloride levels and all-cause mortality remained significant. Specifically, the fourth quartile of serum chloride levels was associated with a significantly increased risk of mortality compared to the first quartile (90-day: HR = 0.54, 95%CI 0.41–0.71; 365-day: HR = 0.52, 95%CI 0.42–0.66). Furthermore, there was an evident increasing trend in the risks of 90-day and 365-day mortality with higher quartiles of serum chloride levels (*P* for trend < 0.001).

**Table 3:** Association between serum chloride and 90-day and 365-day all-cause mortality in COPD critically ill patients.

	Model 1		Model 2		Model 3		Model 4	*P*
	HR (95% CI)	*P*	HR (95% CI)	*P*	HR (95% CI)	*P*	HR (95% CI)	
90d-mortality								
Serum Chloride (continuous)	0.99 (0.97, 1.00)	0.020	0.98 (0.97, 0.99)	0.003	0.98 (0.97, 0.99)	< 0.001	0.96 (0.94, 0.98)	< 0.001
Serum Chloride (categorical)								
Q1	Ref		Ref		Ref		Ref	
Q2	0.66 (0.53, 0.81)	< 0.001	0.65 (0.52, 0.80)	< 0.001	0.66 (0.53, 0.81)	< 0.001	0.64 (0.51, 0.80)	< 0.001
Q3	0.60 (0.49, 0.74)	< 0.001	0.59 (0.48, 0.73)	< 0.001	0.61 (0.49, 0.75)	< 0.001	0.60 (0.47, 0.77)	< 0.001
Q4	0.72 (0.59, 0.89)	0.002	0.67 (0.54, 0.82)	< 0.001	0.64 (0.52, 0.78)	< 0.001	0.54 (0.41, 0.71)	< 0.001
*P* for trend		0.001		< 0.001		< 0.001		< 0.001
365d-mortality								
Serum Chloride (continuous)	0.98 (0.97, 0.99)	< 0.001	0.97 (0.96, 0.98)	< 0.001	0.97 (0.96, 0.98)	< 0.001	0.96 (0.94, 0.97)	< 0.001
Serum Chloride (categorical)								
Q1	Ref		Ref		Ref		Ref	
Q2	0.61 (0.51, 0.73)	< 0.001	0.60 (0.50, 0.72)	< 0.001	0.60 (0.51, 0 .72)	< 0.001	0.60 (0.49, 0.72)	< 0.001
Q3	0.60 (0.50, 0.71)	< 0.001	0.58 (0.49, 0.69)	< 0.001	0.60 (0.50, 0.72)	< 0.001	0.61 (0.50, 0.75)	< 0.001
Q4	0.64 (0.54, 0.76)	< 0.001	0.59 (0.50, 0.71)	< 0.001	0.57 (0.48, 0.68)	< 0.001	0.52 (0.42, 0.66)	< 0.001
*P* for trend		< 0.001		< 0.001		< 0.001		< 0.001

Model 1: no adjusted.

Model 2: adjusted for age, gender, and weight.

Model 3: adjusted for age, gender, weight, WBC, RBC, platelet, hemoglobin, RDW, hypertension, diabetes, and heart failure.

Model 4: adjusted for age, gender, weight, WBC, RBC, platelet, hemoglobin, RDW, hypertension, diabetes, heart failure, myocardial infarction, CKD, stroke, hyperlipidemia, sodium, potassium, calcium, glucose, PT, PTT, BUN, sCR, SOFA, APS III, SIRS, SAPS II, SBP, and DBP.

HR: hazard ratio; CI confidence interval.

### Non-linear relationship between serum chloride and mortality

**Figure 3:**
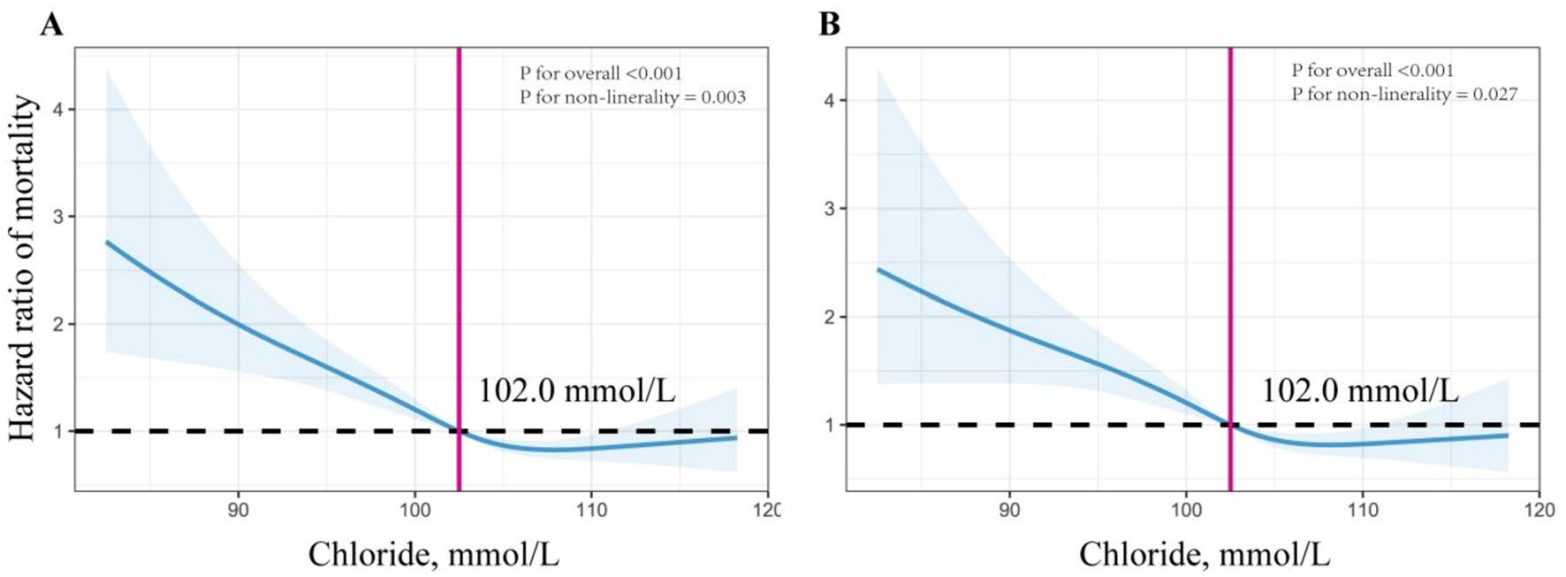
Restricted cubic spline curve for the serum chloride hazard ratio after adjusting for age, gender, weight, WBC, RBC, platelet, hemoglobin, RDW, hypertension, diabetes, heart failure, myocardial infarction, CKD, stroke, hyperlipidemia, sodium, potassium, calcium, glucose, PT, PTT, BUN, sCR, SOFA, APS III, SIRS, SAPS II, SBP, and DBP. (**A**) 365-day mortality, (**B**) 90-day mortality.

To explore the potential nonlinear relationship between serum chloride levels and outcomes, an RCS model was utilized (Fig. 3). The results of the analysis showed a progressive reduction in the risk of death after 90 and 365 days with increasing serum chloride levels and a nonlinear relationship between serum chloride and mortality (*P* for non-linearity < 0.05). However, it is important to note that the magnitude of the decrease in the risk of death diminishes when the serum chloride concentration reaches 102 mmol/L.

### Subgroup analysis

To further examine the robustness of the association between serum chloride concentration and all-cause mortality, subgroup analysis was performed based on various factors, including age, gender, weight, hypertension, diabetes, heart failure, myocardial infarction, CKD, and stroke (Table 4). Subgroup analyses showed that the association between serum chloride levels and all-cause mortality was negative in most of the critically ill patients with COPD (HR < 1). In addition, a significant interaction was observed in the heart failure group in the study with 365-day death as the outcome (*P* for interaction = 0.02).

**Table 4:** Subgroup analysis of serum chloride and 90-day and 365-day mortality in COPD critically ill patients.

	90-day mortality			365-day mortality		
	95% CI	*P*	*P* for interaction	95% CI	*P*	*P* for interaction
Age, years			0.675			0.575
≥ 72	0.95 (0.93, 0.97)	< 0.001		0.95 (0.93, 0.97)	< 0.001	
< 72	0.98 (0.95, 1.01)	0.137		0.97 (0.94, 0.99)	0.008	
Gender			0.731			0.611
Male	0.95(0.93, 0.98)	< 0.001		0.95 (0.93, 0.97)	< 0.001	
Female	0.96 (0.94, 0.99)	0.003		0.97 (0.95, 0.99)	0.002	
Weight, kg			0.889			0.901
< 78.38	0.95 (0.93, 0.97)	< 0.001		0.95 (0.93, 0.97)	< 0.001	
≥ 78.38	0.98 (0.95, 1.00)	0.100		0.97 (0.95, 0.99)	0.006	
Hypertension			0.431			0.977
No	0.96 (0.94, 0.98)	< 0.001		0.96 (0.95, 0.98)	< 0.001	
Yes	0.98 (0.95, 1.02)	0.272		0.96 (0.93, 0.98)	0.002	
Diabetes			0.931			0.877
No	0.95 (0.93, 0.97)	< 0.001		0.95 (0.93, 0.96)	< 0.001	
Yes	1.00 (0.97, 1.04)	0.828		0.99 (0.97, 1.02)	0.688	
Heart failure			0.499			0.202
No	0.96 (0.93, 0.99)	0.003		0.95 (0.93, 0.97)	< 0.001	
Yes	0.96 (0.94, 0.98)	0.002		0.96 (0.94, 0.98)	< 0.001	
Myocardial infarction			0.220			0.098
No	0.96 (0.94, 0.98)	< 0.001		0.96 (0.94, 0.97)	< 0.001	
Yes	0.94 (0.89, 1.00)	0.040		0.96 (0.91, 1.00)	0.062	
CKD			0.424			0.209
No	0.96 (0.94, 0.98)	< 0.001		0.95 (0.93, 0.97)	< 0.001	
Yes	0.97 (0.94, 1.01)	0.123		0.98 (0.96, 1.01)	0.224	
Stroke			0.200			0.200
No	0.96 (0.95, 0.98)	< 0.001		0.96 (0.94, 0.98)	< 0.001	
Yes	0.93 (0.85, 1.01)	0.087		0.95 (0.89, 1.01)	0.123	

Age and weight were grouped according to the median.

The interaction p is calculated according to the multiplicative interaction.

CKD: chronic kidney disease.

### Sensitivity analysis

By excluding participants with missing information, a total of 1969 critically ill COPD patients were included in this study for sensitivity analysis (Table 5). After adjusting for all covariates, the fourth quartile level of serum chloride was associated with significantly lower overall mortality at 90-day (HR = 0.58, 95%CI 0.42–0.78) and 365-day (HR = 0.55, 95%CI 0.42–0.71) compared to the first quartile level.

**Table 5:** Sensitivity analysis between serum chloride and 90-day and 365-day all-cause mortality in critically ill patients with COPD.

	HR (95% CI)	*P*		HR (95% CI)	*P*
90-day mortality			365-day mortality		
Serum Chloride (continuous)	0.97 (0.95, 0.99)	0.001	Serum Chloride (continuous)	0.96 (0.95, 0.98)	< 0.001
Serum Chloride (categorical)			Serum Chloride (categorical)		
Q1	Ref		Q1	Ref	
Q2	0.66 (0.51, 0.85)	0.001	Q2	0.61 (0.49, 0.76)	< 0.001
Q3	0.62 (0.47, 0.82)	< 0.001	Q3	0.63 (0.50, 0.80)	< 0.001
Q4	0.58 (0.42, 0.78)	< 0.001	Q4	0.55 (0.42, 0.71)	< 0.001
P for trend		< 0.001	P for trend		< 0.001

Adjusted for age, gender, weight, WBC, RBC, platelet, hemoglobin, RDW, hypertension, diabetes, heart failure, myocardial infarction, CKD, stroke, hyperlipidemia, sodium, potassium, calcium, glucose, PT, PTT, BUN, sCR, SOFA, APS III, SIRS, SAPS II, SBP, and DBP. HR: hazard ratio; CI confidence interval.

## Discussion

This study represents a novel contribution, as it is the first to investigate the association between serum chloride levels and 90-day and 365-day mortality in critically ill patients with COPD. The findings of this study revealed an inverse relationship between serum chloride levels and overall mortality among COPD critically ill patients, even after accounting for potential confounding factors. Notably, this negative association is an L-shaped pattern, suggesting that as serum chloride levels increased, the mortality decrease slowed down when reaching a concentration of 102 mmol/L. This information provides valuable insights into the potential impact of serum chloride levels on the prognosis of critically ill patients with COPD.

Serum chloride, as the predominant extracellular anion, plays a crucial role in various physiological processes within the human body. There is growing evidence highlighting the close association between elevated serum chloride concentration and organ damage. Numerous studies have reported a correlation between higher serum chloride levels and the development of acute kidney injury^11–13^. Additionally, research has demonstrated that elevated serum chloride levels are linked to a faster decline in the estimated glomerular filtration rate^14^. Moreover, in a study involving patients with Parkinson's disease, a negative association was found between serum chloride ion concentration and the presence of movement impairment, even after adjusting for potential confounding factors^15^. This highlights the potential impact of serum chloride levels on neurological functioning. In relation to the respiratory system, a nationwide survey conducted in the United States revealed an association between higher serum chloride levels and higher forced expiratory volume in 1 s (FEV1), an important measure of lung function^16^. These findings indicate the significance of serum chloride ion levels in respiratory health. Taken together, these studies provide valuable insights into the multifaceted implications of serum chloride levels across different organ systems and underscore the importance of monitoring and understanding their potential effects on overall health and disease outcomes.

In recent years, researchers have increasingly focused on studying the relationship between serum chloride levels and patient prognosis, particularly mortality. Numerous studies have already demonstrated a correlation between increased serum chloride levels and decreased mortality in various populations with different characteristics. For instance, research has shown that lower serum chloride levels are associated with a higher risk of mortality in patients with hypertension^17,18^. Furthermore, a multicenter study involving patients with acute decompensated heart failure found an independent inverse relationship between serum chloride levels upon admission and mortality^5^. Similar negative correlations between serum chloride levels and mortality have been observed in populations such as peritoneal dialysis patients, patients with liver cirrhosis, and patients with acute kidney injury^19^. These findings suggest that serum chloride, as a pervasive anion, plays a vital predictive role in prognosis studies across populations. Consistent with previous research findings, our study also revealed a negative association between serum chloride levels and all-cause mortality in critically ill COPD patients. Notably, our study identified a potential saturated threshold, beyond which the association became less pronounced. These results strengthen the existing evidence on the prognostic significance of serum chloride levels in various patient populations.

Considering the specific characteristics of different patient groups, we conducted a stratified analysis and found that the majority of variables did not significantly modify the association between serum chloride levels and overall mortality in critically ill COPD patients. This suggests that the research results hold value and can be generalized to a broader population. Furthermore, our analysis revealed an interaction effect in patients with comorbid heart failure, which contrasts with previous studies. While the negative association between serum chloride levels and overall mortality was stronger in patients without heart failure, it is crucial to note that in patients with heart failure, the direction of this association aligns with prior research.

Although our study provided insight into the relationship between serum chloride levels and mortality in critically ill patients with COPD by utilizing a large amount of data from the MIMIC-IV database, several shortcomings remain. Firstly, the retrospective nature of this study and the use of data from only a single MIMIC-IV database may limit the general applicability of the findings, and future studies still require data from multiple centers or prospective cohorts to enhance the generalizability and extrapolation of our study. Second, although we controlled for potential confounders by using multiple adjustment models, there may still be unidentified or unmeasured variables that may be associated with serum chloride levels or mortality, and therefore we should be mindful of the applicability of the findings. Finally, although we found an association between serum chloride levels and mortality in critically ill patients with COPD, this association is not directly interpretable as a causal relationship and further studies are still needed to explore the potential mechanisms and possible causal pathways between the two in the future.

## Conclusion

Our study provides evidence of an L-shaped association between serum chloride levels and 90-day and 365-day all-cause mortality in critically ill patients with COPD. This association persisted even after adjusting for potential confounding factors.

### Supplementary Information

Supplementary Table 1.

## Data Availability

This study analyzed publicly available datasets. These data can be found at the following URL: https://physionet.org/content/mimiciv/2.2/.
