# Supplementary material for: L-shaped association between serum chloride levels with 90-day and 365-day all-cause mortality in critically ill patients with COPD: a retrospective cohort study
Source: Sci Rep. 2024 Jul 10;14:15900. doi: 10.1038/s41598-024-67008-7 (PMC11236995; doi:10.1038/s41598-024-67008-7)
Supplement: Supplementary file 1 — Supplementary Table 1. [file 41598_2024_67008_MOESM1_ESM.docx]

| Supplementary Table 1. Missing data of the variables included in this study | |
| --- | --- |
|  | Complete rate |
| Age, years | 1 |
| Gender, n (%) | 1 |
| Weight, kg | 0.993 |
| WBC, K/µL | 0.996 |
| RBC, K/µL | 0.996 |
| Platelet, K/µL | 0.996 |
| Hemoglobin, g/dL | 0.996 |
| RDW, % | 0.996 |
| Sodium, mmol/L | 1 |
| Potassium, mmol/L | 0.998 |
| Calcium, mmol/L | 0.957 |
| Glucose, mmol/L | 0.997 |
| PT, sec | 0.872 |
| PTT, sec | 0.868 |
| BUN, mg/dl | 1 |
| Scr, mg/dl | 1 |
| SOFA | 1 |
| APS III | 1 |
| SIRS | 1 |
| SAPS II | 1 |
| SBP, mmHg | 0.926 |
| DBP, mmHg | 0.926 |
| Hypertenison, n (%) | 1 |
| Diabetes, n (%) | 1 |
| Heart failure, n (%) | 1 |
| Myocardial infarction, n (%) | 1 |
| CKD, n (%) | 1 |
| Stroke, n (%) | 1 |
| Hyperlipidemia, n (%) | 1 |
